# Myosin-X knockout is semi-lethal and demonstrates that myosin-X functions in neural tube closure, pigmentation, hyaloid vasculature regression, and filopodia formation

**DOI:** 10.1038/s41598-017-17638-x

**Published:** 2017-12-11

**Authors:** Ernest G. Heimsath, Yang-In Yim, Mirna Mustapha, John A. Hammer, Richard E. Cheney

**Affiliations:** 1 Department of Cell Biology and Physiology and Lineberger Comprehensive Cancer Center, School of Medicine, University of North Carolina at Chapel Hill, Chapel Hill, NC 27599 USA; 2 Cell Biology and Physiology Center, National Heart, Lung and Blood Institute, National Institutes of Health, Bethesda, MD 20892 USA; 3 Department of Otolaryngology, Stanford University School of Medicine, Palo Alto, CA 94305 USA

## Abstract

Myosin-X (*Myo10*) is an unconventional myosin best known for its striking localization to the tips of filopodia. Despite the broad expression of *Myo10* in vertebrate tissues, its functions at the organismal level remain largely unknown. We report here the generation of KO-first (*Myo10* ^*tm1a/tm1a*^), floxed (*Myo10* ^*tm1c/tm1c*^), and KO mice (*Myo10* ^*tm1d/tm1d*^). Complete knockout of *Myo10* is semi-lethal, with over half of homozygous KO embryos exhibiting exencephaly, a severe defect in neural tube closure. All *Myo10* KO mice that survive birth exhibit a white belly spot, all have persistent fetal vasculature in the eye, and ~50% have webbed digits. *Myo10* KO mice that survive birth can breed and produce litters of KO embryos, demonstrating that *Myo10* is not absolutely essential for mitosis, meiosis, adult survival, or fertility. KO-first mice and an independent spontaneous deletion (*Myo10* ^*m1J/m1J*^) exhibit the same core phenotypes. During retinal angiogenesis, KO mice exhibit a ~50% decrease in endothelial filopodia, demonstrating that *Myo10* is required to form normal numbers of filopodia *in vivo*. The *Myo10* mice generated here demonstrate that *Myo10* has important functions in mammalian development and provide key tools for defining the functions of *Myo10 in vivo*.

## Introduction

Myosin-X (*Myo10*) is an unconventional myosin expressed in most vertebrate tissues, including brain, testis, kidney, and endothelia^1^. Despite intense interest in its cellular and biophysical properties^2,3^, little is known regarding the organismal functions of *Myo10*. Mutant mice have been crucial for understanding the functions of other myosins and their roles in human disease, as illustrated by *Myo5a* in *dilute lethal* mice and human Griscelli syndrome^4^, *Myo6* in *Snell’s Waltzer* mice and human nonsyndromic deafness^5^, *Myo7a* in *Shaker-1* mice and human Usher 1b deaf-blindness syndrome^6,7^, and *Myo15a* in *Shaker-2* mice and human deafness DFNB3^8,9^. To fully define the functions of *Myo10*, it is thus essential to generate *Myo10* knockout (KO) mice and determine their phenotype.

*Myo10* is a member of a phylogenetically ancient group of myosins characterized by one or more MyTH4-FERM domains (Myosin Tail Homology 4 and band 4.1, Ezrin, Radixin, Merlin). The MyTH4-FERM myosins are strongly associated with protrusions based on actin bundles, such as filopodia, microvilli, and inner ear stereocilia^10,11^. Of the four MyTH4-FERM myosins expressed in humans, *Myo7a* is expressed in several tissues and localizes to inner ear stereocilia^12^, *Myo7b* is expressed in transporting epithelia and localizes to the tips of microvilli^13^, *Myo15a* is expressed in the inner ear and localizes to the tips of stereocilia^14^, and *Myo10* is expressed in most cells and localizes to the tips of filopodia^3^.

The heavy chain of full-length mouse *Myo10* is encoded by 41 exons that span 191 kb on chromosome 15. *Myo10* contains 2062 aa (amino acids) that form a head, neck, and tail. The head domain binds to actin filaments and hydrolyzes ATP to generate force and movement^3^. The neck consists of a light chain binding domain with 3 IQ motifs, each of which binds to a calmodulin or calmodulin-like light chain. The tail domain begins with a stable α-helix^15^ followed by a short α-helical region that can dimerize by forming an anti-parallel coiled coil^2,16^. The remainder of the tail includes a PEST region, 3 Pleckstrin Homology (PH) domains, and a MyTH4-FERM domain^1^. The second PH domain binds to the important signaling lipid PI(3,4,5)P_3_, and activation of PI3 kinase is thought to recruit *Myo10* monomers to the plasma membrane, triggering them to form mechanochemically active dimers^17–19^. The MyTH4 domain can bind to microtubules, allowing *Myo10* to act as a motorized link between actin filaments and microtubules^20–22^. The FERM domain can bind to the cytoplasmic domains of several cell surface proteins, including β-integrins^23^ and the netrin receptors DCC (Deleted in Colorectal Cancer) and neogenin^24^. Neogenin is also a coreceptor for Bone Morphogenic Proteins (BMP)^25^, and *Myo10* is upregulated by BMP and functions in BMP signaling^26,27^.

Although most cells and tissues appear to express only the ~237 kD full-length *Myo10*, neural stem cells, neurons, and astrocytes express both full-length and a ~165 kD, “headless” form of *Myo10* ^28,29^. This work showed that use of an alternative transcription start site located ~8.6 kb into intron 19 generates a headless specific exon consisting entirely of 5′ UTR and that splicing this to exons 20–41 generates a transcript for headless *Myo10*. This headless transcript (corresponding to NCBI reference sequence XM_192774.2) encodes a protein expected to initiate at M644 and thus lack most of the motor domain, but is otherwise identical to full-length *Myo10*. The NCBI predicts that two other alternative transcription start sites are located ~13.9 and ~29.5 kb into intron 19 and generate two additional transcripts for headless *Myo10* (XM_006520025.3 and XM_006520024.3). The first encodes a headless protein that is identical to the headless protein described above and corresponds to aa 644–2062 of full-length *Myo10*; the second is identical except that it adds a single M preceding aa 644–2062. Headless *Myo10* does not induce filopodia and has been hypothesized to act as a natural dominant negative and/or as a scaffold that interacts with *Myo10* binding partners such as PIP_3_, microtubules, and β-integrins^29,30^.

Full-length *Myo10* is strongly associated with filopodia^3^, finger-like protrusions thought to function in migration, adhesion, and signaling^31,32^. In cell culture, overexpressing full-length *Myo10* increases filopodia number, whereas inhibiting *Myo10* decreases filopodia number^33–36^. Extremely faint particles of GFP-*Myo10* within filopodia move rapidly towards the tip at rates of ~600 nm/s^37,38^, leading to the hypothesis that full-length *Myo10* powers the intrafilopodial transport of candidate cargo molecules like integrins. *In vitro* studies with *Myo10* indicate that it is specialized to move on parallel actin bundles and can move processively at rates of over ~600 nm/s^2,39^. *Myo10* also functions in microtubule-dependent processes like spindle orientation^20,40,41^. Growing evidence indicates that *Myo10* has important roles in cancer biology. Specifically, *Myo10* is upregulated in several cancers, is a key component of invadopodia, and inhibiting it suppresses invasion^42–45^.

By generating mice with combined loss of full-length and headless *Myo10*, we show that complete loss of *Myo10* is semi-lethal, with over half of null embryos exhibiting exencephaly. *Myo10* null mice that survive birth exhibit several abnormalities, and demonstrate that *Myo10* is required to form normal numbers of filopodia *in vivo*. Together this work shows that *Myo10* has important functions in mammalian development in neural tube closure, pigmentation, and regression of the fetal vasculature of the eye.

## Results

### Generation of *Myo10* KO-first, floxed, and KO mice

**Figure 1:**
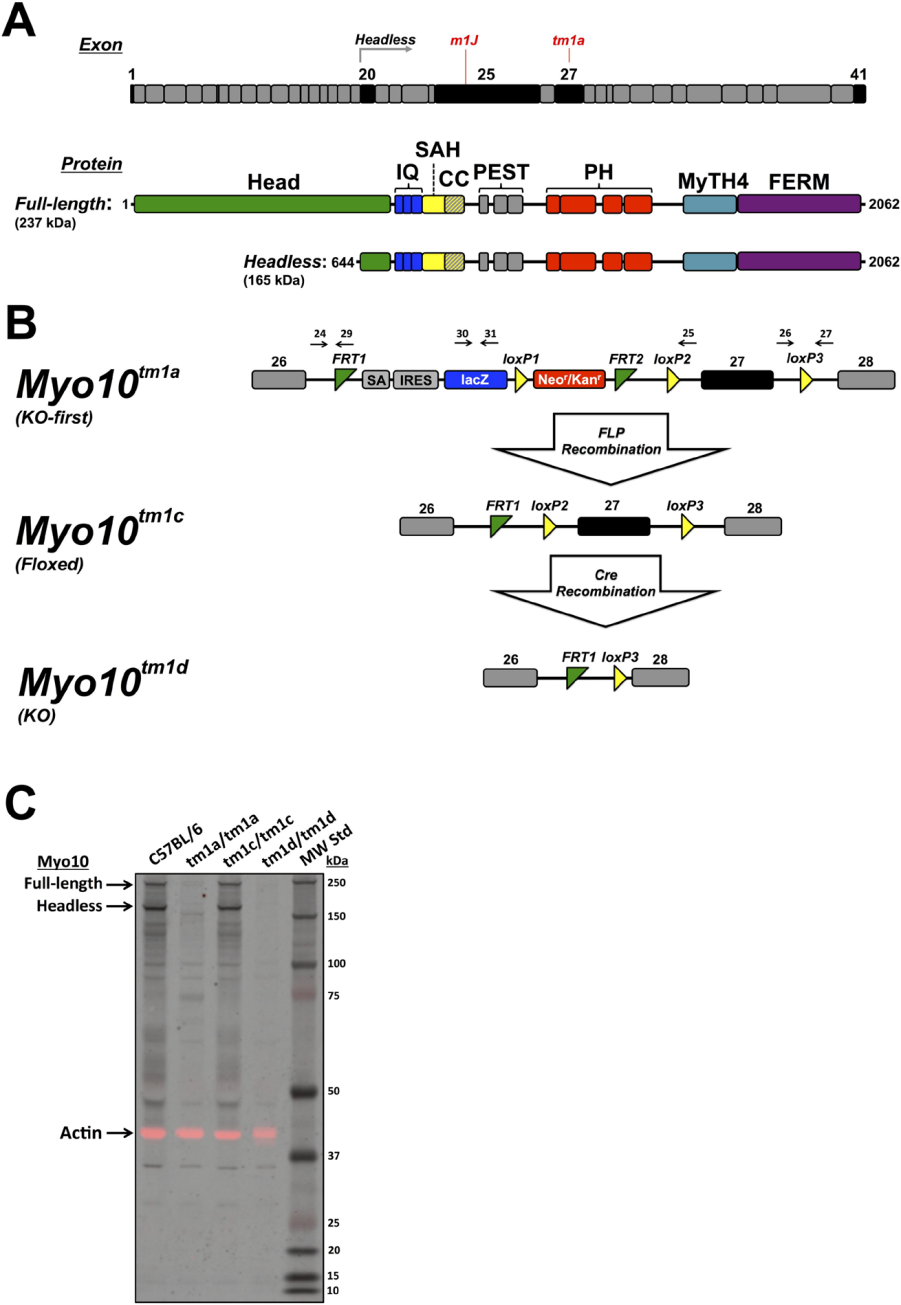
Generation of *Myo10* KO mice. **(A)** Aligned bar diagrams showing exon boundaries relative to the protein domain structure of full-length and headless *Myo10*. The gene for full-length *Myo10* in *Mus musculus* is located on chromosome 15 and contains 41 exons (NCBI Gene ID: 17909). Transcripts for headless *Myo10* are produced by use of three alternative transcription start sites located in intron 19 followed by splicing to exons 20–41. The *Myo10* ^*tm1a*^ mutation targets exon 27 while the spontaneous *Myo10* ^*m1J*^ mutation is due to an 8 bp deletion in exon 25. Exons of particular interest are shaded black. The protein domain structure includes a myosin motor domain (Head), IQ motifs (IQ), stable α-helix (SAH), coiled coil (CC), PEST region, Pleckstrin homology (PH) domains, and a MyTH4-FERM domain. **(B)** Design of the *Myo10* ^*tm1a*^ *, Myo10* ^*tm1c*^, and *Myo10* ^*tm1d*^ alleles. *Myo10* expression in the KO-first allele (*Myo10* ^*tm1a*^) is disrupted due to insertion of a KO first cassette that includes a strong splice acceptor (SA), a stop codon, an internal ribosome entry site (IRES), and LacZ upstream of exon 27. Because the KO-first cassette is flanked by FRT sites, it can be excised by FLP recombinase, restoring *Myo10* expression and generating the conditional *Myo10* ^*tm1c*^ allele. Because exon 27 is flanked by loxP sites, exposure to Cre recombinase deletes exon 27 and generates the *Myo10* ^*tm1d*^ KO allele. Black arrows indicate genotyping primers as described in the Methods. **(C)** Immunoblot of whole brain lysates from P5 mice. As expected, both full-length and headless *Myo10* are detected in the wild-type C57BL/6 and neither is detected in the KO-first *Myo10* ^*tm1a/tm1a*^. Expression of full-length and headless is restored in *Myo10* ^*tm1c/tm1c*^, and neither is detected in the *Myo10* ^*tm1d/tm1d*^ KO. Note that at these exposure settings numerous faintly stained bands below the molecular weights of full-length and headless *Myo10* are visible in the C57BL/6 sample and that most of these putative breakdown products are absent in the KO-first *Myo10* ^*tm1a/tm1a*^ and *Myo10* ^*tm1d/tm1d*^ KO samples. Some non-specifically stained bands are also visible, such as the faint band just below 37 kDa. The signal from the same blot stained with anti-actin (43 kDa) is shown in the red channel as a loading control and has been overlaid onto the black and white *Myo10* signal.

To generate mice that completely lack all forms of *Myo10*, we began by obtaining C57BL/6 derived embryonic stem cells from the Knockout Mouse Project (KOMP) that target exon 27 of *Myo10* with a KO-first cassette (www.komp.org; *Myo10* ^*tm1a(KOMP)Wtsi*^). The KO-first cassette contains a strong splice acceptor site and stop codon upstream of exon 27 that are expected to truncate full-length *Myo10* and the three headless transcripts after aa 1190 (Fig. 1A,B). The KO-first cassette also contains an internal ribosomal entry site (IRES) upstream of lacZ to allow X-gal labeling of cells that express full-length or headless *Myo10*. Because the KO-first cassette is flanked by FRT sites, exposing the *Myo10* ^*tm1a*^ allele to Flp recombinase is expected to excise the KO-first cassette, which should restore *Myo10* expression and generate the conditional *Myo10* ^*tm1c*^ allele. Because exon 27 is flanked by loxP sites, exposure of the *Myo10* ^*tm1c*^ allele to Cre recombinase is expected to delete exon 27 and generate a true genomic KO allele (*Myo10* ^*tm1d*^).

To obtain germline transmission of the *Myo10* ^*tm1a*^ allele on a C57BL/6 background, the *Myo10* ^*tm1a*^ ES cells were injected into blastocysts and the resulting chimeras were bred with albino C57BL/6 mice. Offspring carrying the *Myo10* ^*tm1a*^ allele were crossed with C57BL/6 mice expressing FLPo recombinase to excise the KO-first cassette and generate the floxed *Myo10* ^*tm1c*^ allele on a C57BL/6 background. *Myo10* ^*tm1c*^ mice were then crossed with a global Cre-deleter strain (β-actin Cre) on a C57BL/6 background to delete exon 27 and generate the *Myo10* ^*tm1d*^ KO allele. Mice carrying the *Myo10* ^*tm1d*^ allele were then bred to one another and maintained as a germline KO on the C57BL/6 background. As expected, immunoblots of whole brain from P5 pups showed that control mice expressed both full-length and headless *Myo10*, while neither was detected in the *Myo10* ^*tm1a/tm1a*^ KO-first. Removal of the KO-first cassette produced the conditional *Myo10* ^*tm1c/tm1c*^ and restored expression of full-length and headless *Myo10*, while neither form of *Myo10* was detected in the *Myo10* ^*tm1d/tm1d*^ KO (Fig. 1C).

### *Myo10* KO mice have ~60% incidence of exencephaly

**Figure 2:**
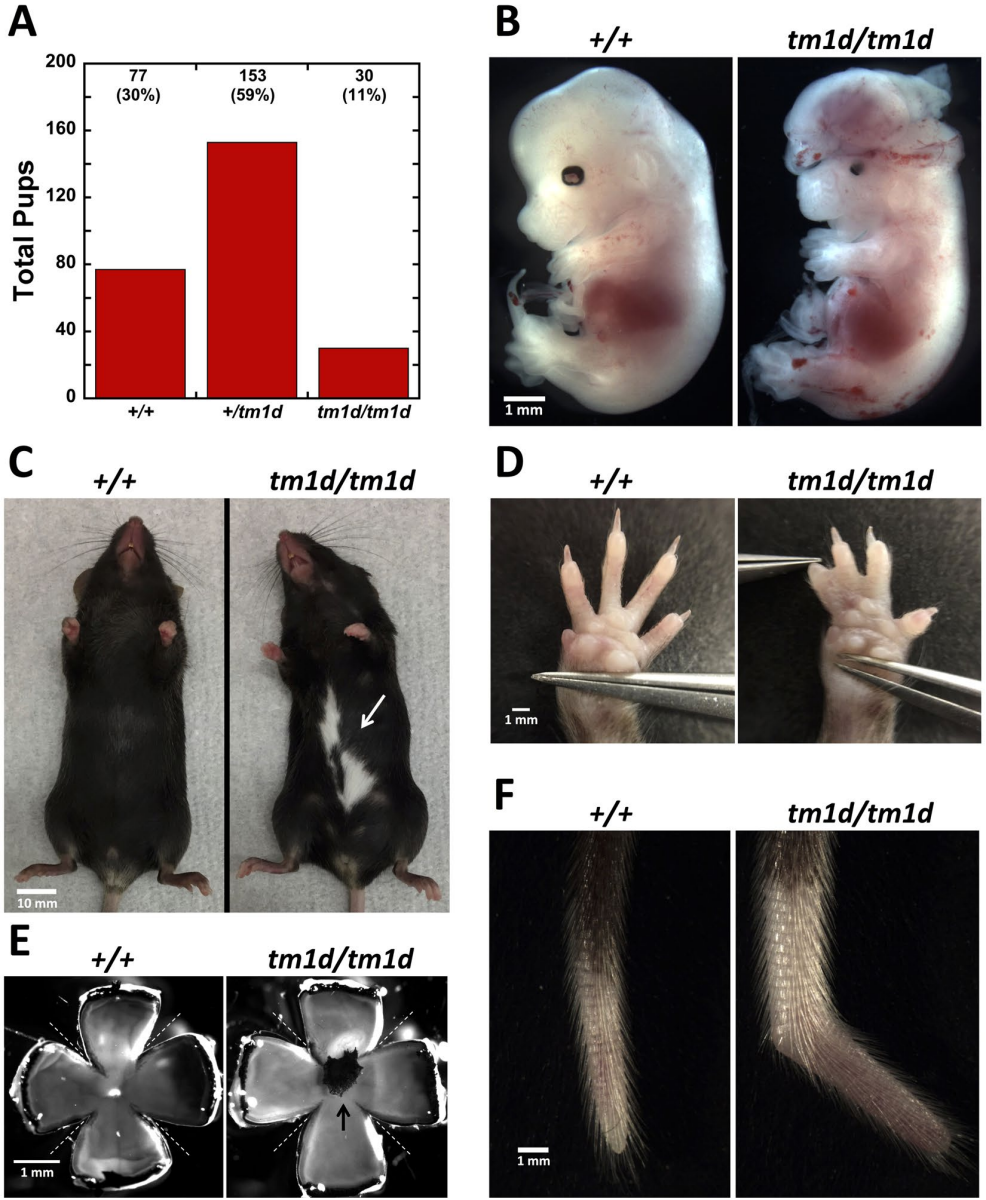
*Myo10* ^*tm1d/tm1d*^ KO mice have a white belly spot and persistent hyaloid vasculature plus a high frequency of exencephaly, webbed digits, and other abnormalities. **(A)** Total number of pups obtained for each genotype from heterozygous matings showing that homozygous nulls are obtained at less than half the expected number (n = 42 litters, 260 pups total, mean litter size 6.1). **(B)** E14.5 embryos showing a normal *Myo10* ^+/+^ control and an exencephalic *Myo10* ^*tm1d/tm1d*^ littermate. Note that the *Myo10* ^*tm1d/tm1d*^ embryo also exhibits microphthalmia**. (C)** Adult mice showing an example of the white belly spot observed in all *Myo10* ^*tm1d/tm1d*^ mice (white arrow). **(D)** Left forepaws from adult mice showing an example of the webbed digits observed in ~50% of *Myo10* ^*tm1d/tm1d*^ mice. **(E)** Eyecups from 6-week-old mice showing a control eye from a wild-type mouse and an example of the pigmented mass present in *Myo10* ^*tm1d/tm1d*^ mice (black arrow). For each eye, the cornea, iris and lens were dissected away and then four incisions were made in each eyecup (dotted lines) to allow the lens to be carefully removed and the eyecup to be partially flattened. **(F)** Tails from adult mice showing an example of a kinked tail in a *Myo10* ^*tm1d/tm1d*^ mouse. Kinked tails were present in most *Myo10* ^*tm1d/tm1d*^ mice, but were often much more subtle.

When heterozygous *Myo10* ^+*/tm1d*^ mice were crossed with one another, pups were obtained at the ratio of 1 *Myo10* ^+/+^: 2 *Myo10* ^+*/tm1d*^: 0.4 *Myo10* ^*tm1d/tm1d*^ (Fig. 2A). This differs from the expected Mendelian ratio of 1:2:1 in that *Myo10* ^*tm1d/tm1d*^ null pups were obtained only ~40% as often as expected (30 *Myo10* ^*tm1d/tm1d*^ null pups versus 77 *Myo10* ^+/+^ wild type). *Myo10* ^+*/tm1d*^ heterozygotes, however, were obtained at the expected 2:1 ratio relative to *Myo10* ^+/+^ homozygotes and appeared indistinguishable from them, thus demonstrating that one copy of the *Myo10* gene is sufficient for normal development. The lower than expected number of *Myo10* ^*tm1d/tm1d*^ pups led us to suspect that ~60% of *Myo10* ^*tm1d/tm1d*^ embryos might undergo embryonic or perinatal lethality and not survive to be counted as pups. To test this, we dissected pregnant *Myo10* ^+/tm1d^ (n = 4) and *Myo10* ^*tm1d/tm1d*^ (n = 2) mice that had been mated to *Myo10* ^*tm1d/tm1d*^ males and examined *Myo10* ^*tm1d/tm1d*^ embryos between E12.5 and E17.5. Strikingly, 15 of the 22 (68%) *Myo10* ^*tm1d/tm1d*^ embryos exhibited exencephaly (Fig. 2B), a severe defect in neural tube closure that is incompatible with survival after birth^46^. Because over half of *Myo10* ^*tm1d/tm1d*^ embryos were exencephalic, these results reveal that *Myo10* has important functions in neural tube closure, and that combined loss of full-length and headless *Myo10* is semi-lethal.

### *Myo10* KO mice have a white belly spot and a high frequency of syndactyly

We next investigated the phenotype of the ~40% of *Myo10* ^*tm1d/tm1d*^ mice that survived birth. The average weight at weaning (P21) was 7.6 g ± 2.1 for *Myo10* ^*tm1d/tm1d*^ (n = 9), which is slightly smaller than the 9.8 g ± 1.0 for *Myo10* ^+/+^ (n = 4, p = 0.026) and 9.9 g ± 1.1 for *Myo10* ^+*/tm1d*^ (n = 9, p = 0.01). A similar difference was also observed at P28 (Supplementary Figure 1). KO pups were able to nurse, feed, and mature into adults. Strikingly, 100% of the *Myo10* ^*tm1d/tm1d*^ mice exhibited a white spot on the belly instead of the uniform black coat expected for C57BL/6 mice (Fig. 2C). Additional pigmentation defects, such as a white spot on the back, were observed in a few cases. The belly spot was noticeable as soon as black pigmentation developed elsewhere and provided a simple visual screen to confirm the PCR genotyping of *Myo10* null mice. ~50% of *Myo10* ^*tm1d/tm1d*^ mice exhibited webbed digits, a form of syndactyly, with the webbed digits usually affecting the forelimb (Fig. 2D). Approximately 75% of *Myo10* ^*tm1d/tm1d*^ mice exhibited a visible kink near the tip of the tail (Fig. 2F). 53% of the homozygous *Myo10* ^*tm1d/tm1d*^ mice obtained were male, and *Myo10* ^*tm1d/tm1d*^ homozygotes were able to develop into mature adults that could mate with one another to produce *Myo10* ^*tm1d/tm1d*^ embryos and pups. This demonstrates that meiosis and mitosis can occur in the complete absence of *Myo10*, and that embryonic development can occur in the absence of maternal *Myo10*. Young adult *Myo10* ^*tm1d/tm1d*^ mice did not exhibit obvious movement defects like circling or shaking and responded to a hand clap by flicking their ears (Preyer reflex), indicating that hearing and balance are grossly intact at this age. The oldest *Myo10* ^*tm1d/tm1d*^ mice have survived over a year and a half without obvious morbidity or mortality. Together these results demonstrate that *Myo10* has important roles in mammalian development, but is not strictly essential for adult survival or fertility.

### *Myo10* KO mice have persistent hyaloid vasculature

Dissection of eyes from adult mice followed by careful removal of the lens revealed that a black, pigmented mass was present bilaterally in all 11 *Myo10* ^*tm1d/tm1d*^ mice tested but none of 14 *Myo10* ^+/+^ *or* *Myo10* ^+/tm1d^ mice (Fig. 2E) The mass was typically attached to the optic disc by a thin strand and extended through the vitreous to form a plexus on the posterior surface of the lens. In some eyes, the retrolental mass appeared to have integrated into the retina and disrupted the retina’s normal morphology. The position and morphology of the retrolental mass indicate that it is persistent hyaloid vasculature. This condition is also known as persistent fetal vasculature (PFV) or persistent hyperplastic primary vitreous (PHPV), and results from a hyperplasia and persistence of a normally transient fetal vasculature that perfuses the vitreous during development^47^. The hyaloid vasculature originates at the optic disk as the stalk of the hyaloid artery and includes numerous branches, some of which extend through the periphery of the vitreous while others form a plexus on the posterior surface of the lens. The hyaloid vasculature in mouse begins forming at approximately E12.5 as the optic fissure closes, and is normally resorbed over the first few postnatal weeks as the retinal vasculature grows out onto the surface of the retina and matures. In humans, PFV is responsible for 5% of childhood blindness and is associated with increased incidence of several other ocular abnormalities including microphthalmia (a small eye)^47^. When a series of 11 *Myo10* ^*tm1d/tm1d*^ E12.5-E14.5 embryos were examined for major eye defects, 3/11 exhibited severe microphthalmia and 2/11 exhibited anophthalmia (absence of an eye).

**Figure 3:**
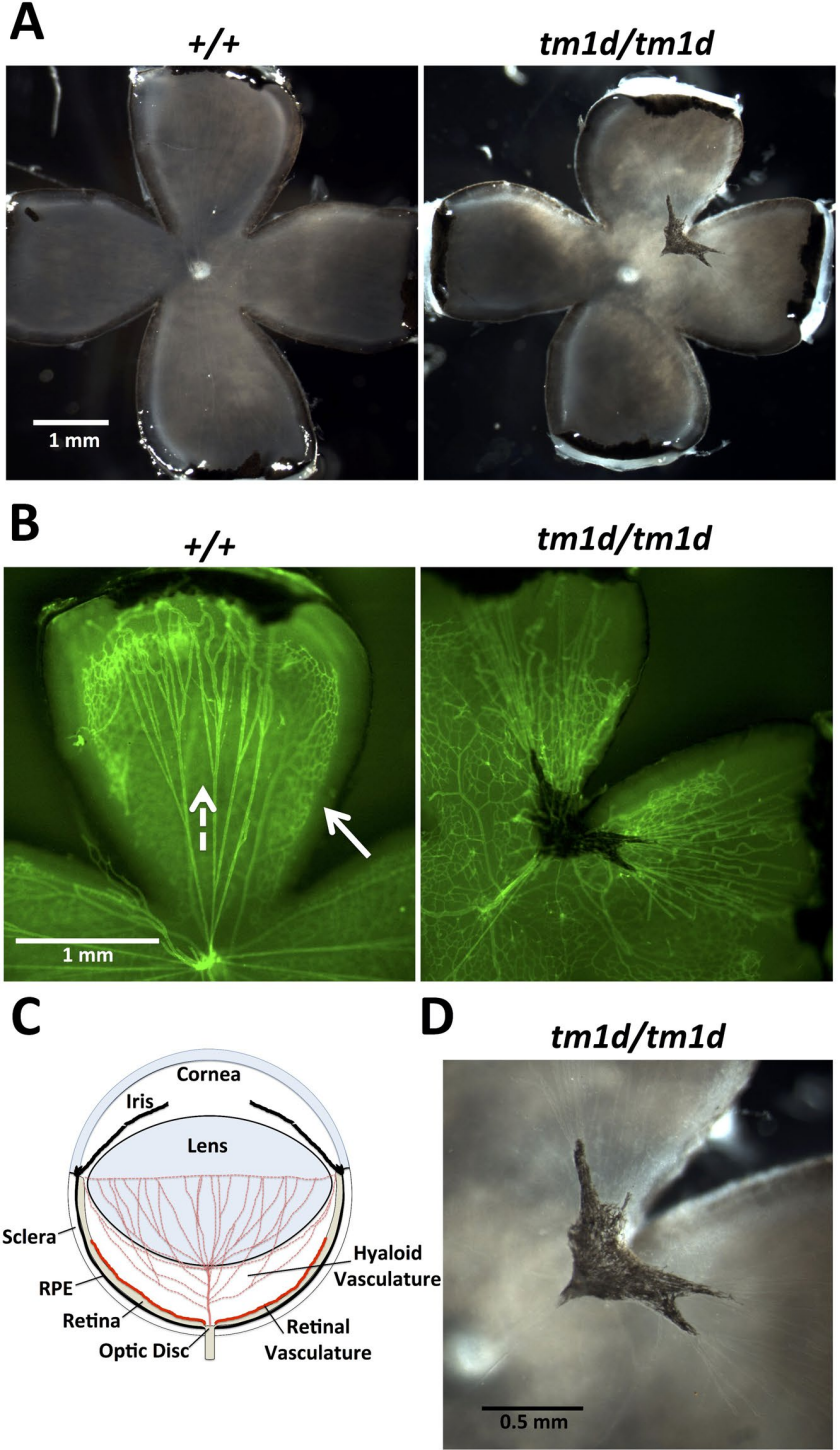
Eyes from *Myo10* ^*tm1d/tm1d*^ KO mice have persistent hyaloid vasculature. **(A)** Eyecups from P5 mice showing a control eye from a wild-type mouse and an example of the pigmented persistent hyaloid vasculature present in a *Myo10* ^*tm1d/tm1d*^ mouse. The white spot in the center of each eye is the optic disk. An irregular black mass is located above and to the right of the optic disc in the *Myo10* ^*tm1d/tm1d*^ eye. **(B)** The same eyecups in (**A**) were fluorescently stained with a PECAM-1 antibody (green) to label endothelial cells. In the control at left, several long and relatively straight branches (dotted white arrow) of the hyaloid vasculature can be seen extending from their origin near the optic disk towards the rim of the eyecup. Consistent with their identification as hyaloid vasculature, they are located within the vitreous and slightly above the developing retinal vasculature, which forms reticulum on the surface of the retina (solid white arrow). In the *Myo10* ^*tm1d/tm1d*^ eye, a relatively thick hyaloid artery extends from the optic disk to the pigmented mass, while long and relatively straight branches of the hylaloid vasculature extend outward from arms of the pigmented mass. Because the eyecups curve upwards at their outer edges, the view there is foreshortened. The hyaloid and retinal vasculatures can be best distinguished by enlarging the images on a digital display. **(C)** Schematic cross-section of a normal P5 eye. At this stage the hyaloid vasculature has begun to regress, but still perfuses the vitreous and forms a plexus on the posterior of the lens, while the retinal vasculature is undergoing angiogenesis and extending on the surface of the retina. Illustration created by EGH. **(D)** Close-up view of the pigmented mass in (**A**) and (**B**) showing that it includes a dense plexus of hyaloid vasculature intermixed with pigment cells. The plexus can be seen most clearly after enlargement on a digital display.

To investigate the development of the persistent hyaloid vasculature, we dissected eyes from pups at P5, a time when the hyaloid vasculature would have just started to resorb. The retinal vasculature, on the other hand, would have begun angiogenesis at ~P0 by growing out from the optic disc and by P5 should extend approximately halfway across the retina^48^. Dissection revealed that a pigmented mass (Fig. 3A) was present bilaterally in 17 *Myo10* ^*tm1d/tm1d*^ mice, while no examples were present in any of the eyes from 12 control mice. Immunofluorescence with the endothelial cell marker PECAM-1 (CD31) showed that numerous long and relatively straight hyaloid vessels were present in the control eye at P5; as expected, the hyaloid vessels originated near the optic disk and extended branches towards the ciliary body (Fig. 3B). In the *Myo10* ^*tm1d/tm1d*^ retina, however, the hyaloid artery extended as a thickened stalk from the optic disk into a pigmented mass. The pigmented mass includes a plexus of blood vessels and pigment cells, with long and relatively straight hyaloid vessels extending outward from arms on the mass toward the ciliary body. The presence of the pigmented mass at P5 demonstrates that it forms and becomes pigmented prior to the major regression of hyaloid vessels in control eyes. Pigmented cells were not observed on either the stalk of the hyaloid artery or the hyaloid vessels extending towards the ciliary body, both of which represent potential pathways for pigment cell migration (Fig. 3B,D). Together these experiments demonstrate that a core element of the *Myo10* ^*tm1d/tm1d*^ phenotype is 100% penetrance of persistent hyaloid vasculature that is pigmented and bilateral.

### *Myo10* KO-first mice also exhibit a high incidence of exencephaly, a white belly spot, and retention of the hyaloid vasculature

In addition to determining the phenotype of the definitive germline KO mice, we also investigated the phenotype of the KO-first mice (*Myo10* ^*tm1a/tm1a*^). *Myo10* ^+*/tm1a*^ heterozygotes appeared indistinguishable from controls, but when they were crossed to each other, pups were obtained at the ratio of 1 *Myo10* ^+/+^: 1.5 *Myo10* ^+*/tm1a*^: 0.45 *Myo10* ^*tm1a/tm1a*^ (20 *Myo10* ^+/+^, 30 *Myo10* ^+*/tm1a*^, 9 *Myo10* ^*tm1a/tm1a*^; n = 9 litters). Because *Myo10* ^*tm1a/tm1a*^ homozygotes were obtained only 45% as often as *Myo10* ^+/+^ homozygotes, these results again suggested that loss of *Myo10* was leading to a high frequency of embryonic or perinatal lethality. Consistent with this, examination of *Myo10* ^*tm1a/tm1a*^ E12.5 embryos resulting from 2 homozygous crosses of *Myo10* ^*tm1a/tm1a*^ mice revealed that 50% (9/18) had exencephaly. *Myo10* ^*tm1a/tm1a*^ homozygotes that survived birth grew to adulthood and 100% had a white belly spot. A few also had additional pigmentation defects such as a white spot on the back. 100% of *Myo10* ^*tm1a/tm1a*^ mice whose eyes were dissected (5/5) had persistent hyaloid vasculature that was pigmented and bilateral. Approximately 50% of *Myo10* ^*tm1a/tm1a*^ mice had webbed digits and similar fraction had a kink near the tip of the tail. Importantly, when the KO-first mice were crossed with FLPo mice to delete the KO-first cassette, *Myo10* expression was restored (Fig. 1C) and the resulting *Myo10* ^*tm1c/tm1c*^ mice appeared completely normal. This rescue of the KO-first phenotype provides strong genetic evidence that the phenotypes reported here are specifically due to disruption of *Myo10*.

### *Myo10* is required to form normal numbers of filopodia during retinal angiogenesis

**Figure 4:**
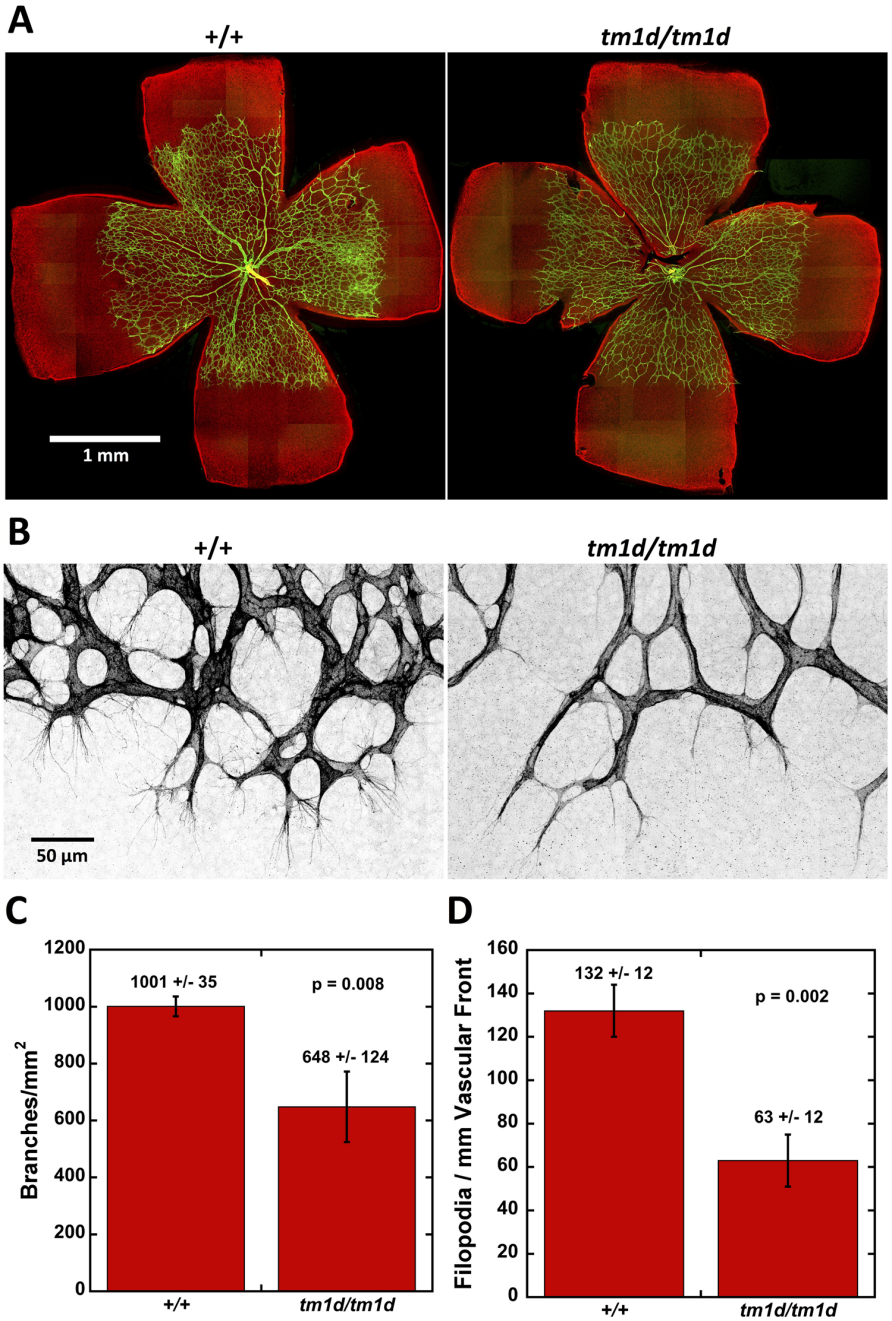
Loss of *Myo10* decreases the number of filopodia during retinal angiogenesis. **(A)** Fluorescence image of flat mounted retinas showing retinal vasculature at P5. Dissected retinas were stained with a PECAM-1 antibody (green) and counterstained with phalloidin (red). The retinal vasculature extended to similar positions in control and *Myo10* ^*tm1d/tm1d*^ eyes. The image represents a stitch of micrographs taken at 20X and is best visualized if the images are enlarged on a digital display. **(B)** High resolution images of the angiogenic expansion front from the P5 retinas in (**A**) showing filopodia radiating from endothelial tips cells. Loss of *Myo10* results in a decreased number of filopodia and leads to a less dense vascular network. Images were captured as Z-stacks at 60X and displayed as maximum projections with the PECAM-1 channel displayed in inverted grayscale to highlight endothelial filopodia. **(C)** Quantification showing that loss of *Myo10* decreases the branch point density/mm^2^ by 32%. **(D)** Quantification showing that loss of *Myo10* decreased the number of filopodia per mm of vascular front by ~50%. N = 3 mice for both (**C**) and (**D**) and error bars denote standard deviation.

Given *in vitro* studies indicating that *Myo10* is a key component of filopodia^3^ we next sought to determine if *Myo10* is required for filopodial formation *in vivo*. Therefore, we turned to the retinal vasculature, where endothelial tip cells generate numerous filopodia during angiogenesis as they grow out across the retina^48,49^. To visualize the endothelial cells and their filopodia, P5 retinas were stained with a PECAM-1 antibody (Fig. 4A). Low power views showed that the retinal vasculature grew similar distances across the retina in both control and *Myo10* ^*tm1d/tm1d*^ retinas (1.34  ± 0.11 mm for 9 controls and 1.25  ± 0.08 for 5 *Myo10* ^*tm1d/tm1d*^, p = 0.09), demonstrating that angiogenic outgrowth can occur in the absence of *Myo10*. The vascular network, however, appeared to be less dense in *Myo10* ^*tm1d/tm1d*^ retinas, and quantification showed that the branches/mm^2^ decreased by over 30% (Fig. 4A,C). To determine if loss of *Myo10* decreased filopodia, we collected Z-stacks of the vascular front at 60x and displayed these as maximum projections. This revealed that the endothelial cells in *Myo10* ^*tm1d/tm1d*^ retinas had far fewer filopodia than controls (Fig. 4B). Quantification showed a ~50% decrease in filopodia per mm along the vascular front (Fig. 4D **)**, thus demonstrating that *Myo10* is required to form normal numbers of filopodia *in vivo*.

### *Myo10*^*m1J/m1J*^ mice exhibit exencephaly, a white belly spot, and retention of the hyaloid vasculature

To conclusively define the phenotype resulting from loss of full-length and headless *Myo10*, we also examined mice carrying an independent mutation (*Myo10* ^*m1J*^) that was recently listed along with 91 other new mouse mutations identified at The Jackson Laboratory^50^. The *Myo10* ^*m1J*^ mutation, which is on a C57BL/6 background, is due to a spontaneous 8 bp deletion (CGACGAGT) in exon 25 and produces a frame shift expected to truncate full-length *Myo10* and all three headless transcripts following aa 948. The brief online description of the *Myo10* ^*m1J*^ mutation (http://www.informatics.jax.org/reference/J:214794) indicates that homozygotes exhibited a white belly spot. Syndactyly on the forelimbs was also noted, and persistent fetal vasculature was visible in the two mice whose eyes were examined. Homozygous *Myo10* ^*m1J/m1J*^ mice were listed as viable, but as having a decreased body size. However, no immunoblots were performed to confirm loss of *Myo10* protein, no data was presented on the proportion of homozygotes obtained, and no mention was made of exencephaly.

**Figure 5:**
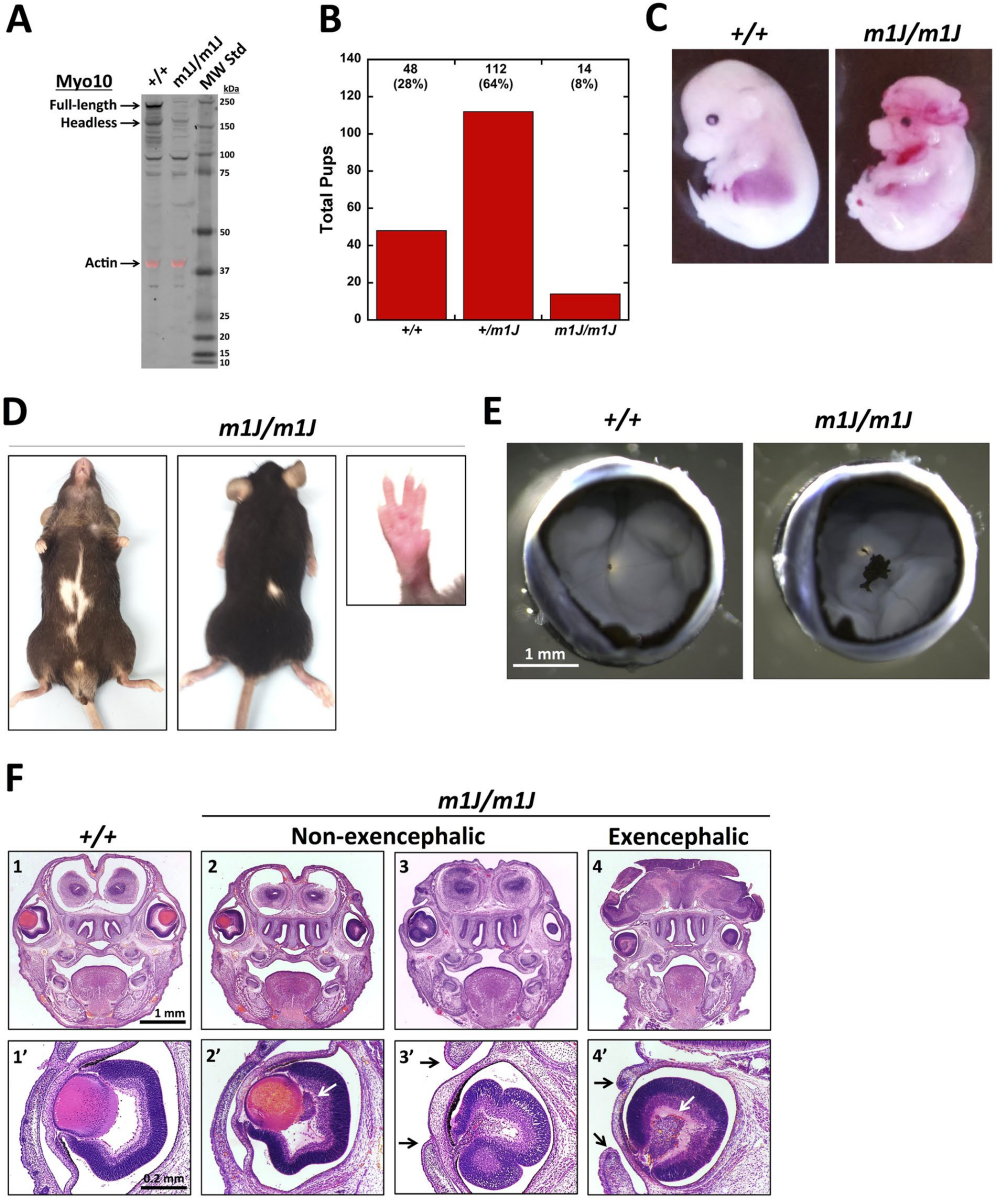
*Myo10* ^*m1J /m1J*^ mice have a white belly spot and persistent hyaloid vasculature plus a high frequency of exencephaly and other abnormalities. **(A)** Immunoblot of whole brain lysates from 1 month old mice showing deletion of full-length and headless *Myo10*. Although bands corresponding to full-length and headless *Myo10* are absent in the *Myo10* ^*m1J /m1J*^ lysate, it should be noted that the *Myo10* antibody shows some non-specific staining, including one band that is slightly larger than headless and another that is near the 100 kDa standard. The signal from the same blot stained with anti-actin (43 kDa) is shown in the red channel as a loading control and has been overlaid onto the black and white *Myo10* signal. **(B)** Total number of pups obtained for each genotype from heterozygous matings showing that *Myo10* ^*m1J /m1J*^ homozygotes were obtained at less than half the expected number (n = 28 litters, 174 pups total; mean litter size 6.2). **(C)** E15.5 embryos showing a *Myo10* ^+/+^ control and an exencephalic *Myo10* ^*m1J /m1J*^ littermate. **(D)** Adult *Myo10* ^*m1J/m1J*^ mouse showing examples of a white belly spot, a white spot on the back, and syndactyly. **(E)** Eyecups from 6-week old mice showing a normal eye from a *Myo10* ^+/+^ mouse and an example of the pigmented mass of persistent hyaloid vasculature present in the eyes of *Myo10* ^*m1J/m1J*^ mice. **(F)** Rostral coronal sections from E16.5 embryos showing a wild-type, two nulls that did not have exencephaly, and one null that did have exencephaly. Note the relatively normal appearance of the brain and cranium in the null embryos without exencephaly and the lack of a cranium and gross defects in brain morphology in the embryo with exencephaly. The null embryo on the left was selected for sectioning in part because it had normal sized eyes while the two embryos on right had microphthalmia. Note that slight differences in the plane of section between the left and right side of the skull make some of the eyes on the right side appear smaller than they actually are. Higher magnification images of sections through the middle of the eyes showing the abnormal accumulations of cells (white arrows) behind the lens and defects in lens development in the two eyes on the right (at this stage the lens should be visible as red stained sphere). Note also that the null eyes in 3′ and 4′ failed to undergo eyelid closure (black arrows), a process that normally occurs between E15.5 and E16.5.

Immunoblots of whole brain from a month-old *Myo10* ^*m1J/m1J*^ mouse show that neither full-length nor headless *Myo10* was detected, demonstrating that the mutation results in complete loss of *Myo10* (Fig. 5A). *Myo10* ^+/m1J^ heterozygotes appeared normal, but when they were mated to one another, pups were obtained in the ratio of 1 *Myo10* ^+/+^: 2.3 *Myo10* ^+*/m1J*^: 0.3 *Myo10* ^*m1J/m1J*^ (Fig. 5B). The much lower than expected number of *Myo10* ^*m1J/m1J*^ pups (14 *Myo10* ^*m1J/m1J*^ nulls versus 48 *Myo10* ^+/+^) again suggested that loss of *Myo10* leads a high level of embryonic or perinatal lethality. Analysis of embryos (E12.5-E17.5) from heterozygous matings revealed that 22/88 (25%) were *Myo10* ^*m1J/m1J*^ homozygotes, indicating that null embryos were present in the expected Mendelian percentage. Examination of the 22 null embryos revealed that 73% had exencephaly (Fig. 5C), 18% had craniorachischisis (a major failure of neural tube closure at the level of the hindbrain and spinal cord), and 9% had a gross developmental defect such as very small size. This demonstrates that a large fraction of *Myo10* ^*m1J/m1J*^ embryos have exencephaly and/or other defects that would be incompatible with survival past birth. Examples of fetuses undergoing resorption were also observed, and 64% of homozygous embryos exhibited blood in the amniotic fluid. Homozygous embryos appeared slightly smaller than controls, even when not affected by exencephaly or other major defects.

Of the 14 homozygous *Myo10* ^*m1J/m1J*^ pups obtained above, 6/14 were male, 14/14 had a white belly spot, 4/14 also had a white spot on the back, 6/14 had webbed digits on one or both forelimbs (Fig. 5D). Kinked tails were not obvious in *Myo10* ^*m1J/m1J*^ mice and were not scored due to the difficulty of defining very mild kinks. Anophthalmia was present in 1/14 of the *Myo10* ^*m1J/m1J*^ mice and 3/14 had severe microphthalmia. All 5 of the *Myo10* ^*m1J/m1J*^ homozygotes whose eyes were dissected at 6 weeks had persistent hyaloid vasculature, and this was pigmented and present bilaterally (Fig. 5E). The average weight at weaning (P21) for *Myo10* ^*m1J/m1J*^ pups was 8.5 ± 1.5 g (N = 7), which is slightly smaller than the 11.3 ± 0.7 g for *Myo10* ^+*/m1J*^ (N = 38, p = 0.00002) and 11.6  ± 0.5 g for *Myo10* ^+/+^ (N = 18; p = 0.00002). Similar results were obtained at P28 (Supplementary Figure 1). *Myo10* ^*m1J/m1J*^ homozygotes that survived birth were able to feed and develop into adults. Young adult homozygotes did not exhibit obvious circling or shaking and responded to a clap by flicking their ears. Homozygotes could successfully mate, and analysis of the 8 E15.5 embryos obtained from a homozygous mating revealed that 1 was very small and appeared to lack a liver while 7 had exencephaly, including one that also had omphalocele (protrusion of abdominal organs outside the abdominal wall). All 8 embryos also exhibited blood in the amniotic sac, although this intra-amniotic hemorrhage may have been secondary to the major developmental defects like exencephaly. Together these results demonstrate that the phenotypes of the spontaneous *Myo10* ^*m1J/m1J*^ mutation are very similar to those of the targeted *Myo10* ^*tm1a/tm1a*^ and *Myo10* ^*m1d/m1d*^ mutations, although the *Myo10* ^*m1J/m1J*^ phenotype may be somewhat more severe.

To further investigate the defects resulting from loss of *Myo10*, hematoxylin and eosin stained sections were prepared from the heads of control and *Myo10* ^*m1J/m1J*^ embryos at E16.5 (Fig. 5F). Rostral coronal sections show relatively normal development of the brain and cranium in *Myo10* ^*m1J/m1J*^ embryos that were not exencephalic, as well as the absence of a cranium and gross defects in brain morphology in an exencephalic embryo. Higher magnification views of *Myo10* ^*m1J/m1J*^ eyes at E16.5 revealed examples of abnormally large numbers of cells behind the lens, as expected for the development of persistent hyaloid vasculature. *Myo10* ^*m1J/m1J*^ eyes also revealed examples of other abnormalities that are often associated with persistent hyaloid vasculature, such as defects in lens formation. We also observed examples of failed embryonic eyelid closure (Figs 5F3′ and 4′), a process that normally occurs at E15.5–16.5 when sheets of eyelid cells extend across the eye and fuse^51^. This defect or delay in eyelid closure was observed both in exencephalic and non-exencephalic *Myo10* ^*m1J/m1J*^ embryos, demonstrating that it is not secondary to exencephaly. Taken together, the results here define the core phenotype resulting from complete loss of full-length and headless *Myo10*. They also demonstrate that *Myo10* has important roles in neural tube closure, pigmentation, regression of the fetal vasculature of the eye, and filopodia formation.

## Discussion

### *Myo10* functions in filopodia formation and angiogenesis *in vivo*

A key feature of *Myo10* deduced from studies in cell culture is its roles in filopodia – *Myo10* induces filopodia, traffics within filopodia, and localizes to filopodial tips^3^. We show here that the angiogenic front in *Myo10* KO retinas exhibits ~50% fewer filopodia than controls, thus demonstrating that *Myo10* functions in filopodia formation *in vivo*. Because we have previously shown that endothelial cells in culture express full-length *Myo10*, and that *Myo10* knockdown decreases filopodia number^26^, it is likely that the decreased filopodia in KO retinas is cell autonomous and due to the loss of the filopodia-promoting activity of full-length *Myo10*. Our results also reveal that some filopodia are able to form in the complete absence of *Myo10*, which demonstrates the existence of a *Myo10* independent pathway for filopodia formation. Our results on filopodia formation *in vivo* are also consistent with recent results showing that macrophages cultured from a different *Myo10* KO mouse exhibit greatly decreased filopodia formation^36^. A description of the KOMP-generated *Myo10* ^*tm2/tm2*^ mouse has not yet been published, but the allele includes a 9.5 kb deletion that disrupts exon 19 and is expected to delete full-length *Myo10* and the first of the three headless transcripts. Although the slender protrusions that form in the absence of *Myo10* have a filopodial morphology, it should be kept in mind that they may differ from normal filopodia. In the future, it will be important to investigate their composition and dynamics and to determine if they lack components reported to interact with *Myo10*, such as β-integrins^23^ and VE-cadherin^52^. Interestingly, knockout of fascin, the major actin bundling protein in filopodia, also decreases filopodia without completely eliminating them^53,54^. The ability of filopodia to form in the complete absence of either their major actin bundling protein or the molecular motor *Myo10* suggests that filopodia formation is a robust process with substantial redundancy.

Because the vasculature in control and *Myo10* KO mice grew out to similar positions on the retina by P5, neither *Myo10* nor a full complement of filopodia were essential for endothelial outgrowth. Loss of *Myo10* did reduce the density of branch points by ~30%. This suggests that *Myo10* and filopodia may be more important for forming branches and cell-cell contacts than for outgrowth *per se*. Somewhat similar results were reported in zebrafish angiogenesis when filopodia formation was completely eliminated using a low dose of the anti-actin drug latrunculin^55^. Under those conditions, developing vessels were still able to grow in the proper direction using lamellipodia, but they exhibited defects in their ability to connect to one another. Loss of *Myo10* may well have additional effects on vascular patterning, and given the importance of angiogenesis in development, cancer, and eye disease, further investigation of the roles of *Myo10* and filopodia in angiogenesis is warranted.

### *Myo10* functions in neural tube closure

In all three strains of null mice, half or more of the embryos exhibited exencephaly. Exencephaly is a severe neural tube defect resulting from failure to close the neural tube at the cranial level^46^. Neural tube defects are a leading cause of human birth defects, affecting 300,000 births per year worldwide^56^. Exencephaly constitutes one third of all neural tube defects and leads to a defect in cranium formation and exposure of the brain to amniotic fluid. In both mouse and human, neural tube closure initiates at the boundary between the developing hindbrain and spinal cord. Failure of this initial closure is associated with mutations in the canonical Wnt-frizzled planar cell polarity pathway and results in craniorachischisis, while failure to close the neural tube at the midbrain or its anterior end results in exencephaly^46^. Because we observed a high incidence of exencephaly and only occasional examples of craniorachischisis, *Myo10* is most crucial for neural tube closure at the cranial level. Given that exencephaly is incompatible with survival after birth and was observed in half or more *Myo10* null embryos, we conclude that exencephaly is the major cause of death in *Myo10* nulls. Most importantly, our results reveal a previously unsuspected requirement for *Myo10* in neurulation.

Exencephaly can result from defects in several processes, including proliferation of neural plate cells, morphogenesis of the neural folds, and failure of the neural folds to contact one another and fuse to form a tube. *Myo10* mRNA is reported to be enriched in the neural plate and cranial neural crest in *Xenopus*, and morpholino-mediated knockdown in *Xenopus* resulted in craniofacial abnormalities^57,58^. Our results conclusively demonstrate that *Myo10* functions in mammalian neural tube closure, but it is not yet clear whether closure requires full-length *Myo10*, headless *Myo10*, or both forms. Headless *Myo10* is expressed in neural stem cells^29^ and could act either on its own or by antagonizing the actions of full-length *Myo10* in processes such as spindle orientation or filopodia formation. Since filopodia-like protrusions have been observed extending from the cranial neural folds and are hypothesized to help the folds contact and fuse^59^, the filopodia-promoting activities of full-length *Myo10* are strong candidates to facilitate the contact and zippering stages of neural tube closure. Knockouts of other pro-filopodial proteins have been reported to result in exencephaly, as illustrated by the *Mena/VASP/Evl* triple KO^60^. No *Mena/VASP/Evl* triple KO mice survived birth, but the embryos were reported to exhibit defects such as intraamniotic hemorrhage and a high frequency of exencephaly. Filopodia are known to be very sensitive to low doses of cytochalasin^61^, and exposure to this anti-actin drug during neural tube closure induces exencephaly^62,63^. In addition to *Myo10*’s activities in filopodia formation and spindle orientation, it could also act in the signaling pathways required for neural tube closure. This is especially true given that *Myo10* binds to neogenin^24^, a receptor for Repulsive Guidance Molecule a (RGMa) and a co-receptor for Bone Morphogenic Proteins (BMP)^25^, and that knockdown of neogenin results in 100% exencephaly^64^, while deletion of RGMa results in 50% exencephaly^65^.

Since most *Myo10* null embryos that succeeded in closing the neural tube developed into viable adults, neural tube closure is the critical point for *Myo10* function during development. It is not clear why the exencephaly is partially penetrant, but variation in genetic background can be ruled out since the *Myo10* ^*m1J/m1J*^ null mice were on a congenic C57BL/6 background. Partial penetrance has recently been shown to be extremely common among many mouse traits^66^ and is generally attributed to stochastic variation in processes such as gene expression and subtle differences in the environment. It is also not clear why some aspects of the phenotype such as the exencephaly appear to differ in their penetrance between the different *Myo10* alleles. Some of this may be attributable to random variation associated with the sample sizes. It is also possible that *Myo10* ^*tm1d/tm1d*^ and *Myo10* ^*m1J/m1J*^ deletions produce trace amounts of truncated *Myo10* that are below our detection threshold for blotting and that the different lengths of *Myo10* they would encode (aa 1–948 versus aa 1–1190) influence the penetrance. Although the work here defines the core *Myo10* phenotype in C57BL/6 mice, it should be kept in mind that the phenotype may differ in other species or genetic backgrounds. In the future, it will be important to determine which forms of *Myo10* are required for neural tube closure, the precise functions of *Myo10* in closure, and if exencephaly in humans is associated with mutations in *Myo10*.

### *Myo10* is required for normal pigmentation

All three *Myo10* null mutations characterized here exhibited 100% penetrance of a white belly spot. Moreover, other pigmentation defects such as a white spot on the back were observed at lower penetrance. White spots on the belly or chest are present in several types of mutant mice, and are also frequently present in cats, dogs, and other domestic animals^67^. Such unpigmented regions are usually due to a defect in the production and/or migration of melanoblasts – neural crest derivatives that must migrate long distances to reach the ventral surface where they differentiate into melanocytes. Migrating neural crest cells are highly filopodial^68^ and mice lacking the filopodia-promoting proteins fascin^69^ or Cdc42 have white belly spots^70^. Since only full-length *Myo10* is detected in mammalian melanocytes^27^, it is likely to act by promoting filopodia and facilitating melanocyte migration.

Full-length *Myo10* and filopodia have also been hypothesized to function in the transfer of melanosomes from melanocytes to skin keratinocytes based on cell culture experiments with human cells^27,71^. In *Myo10* null mice, however, most of the coat remains black and does not exhibit the pigmentary dilution due to defects in melanosome positioning and transfer seen in *dilute*/*Myo5a* ^4^, *ashen*/*Rab27a* ^72^, and *leaden/Mlph* mice^73^. The fact that most of the coat in *Myo10* nulls remains black argues that *Myo10* is not essential for melanosome transfer from melanocytes to keratinocytes *in vivo*, at least in mice.

### *Myo10* is required for regression of the fetal vasculature in the eye

In the three strains of *Myo10* KO mice characterized here, all of the eyes that were examined had persistent hyaloid vasculature, a normally transient vasculature that nourishes the developing eye^74^. PFV is often associated with increased incidence of other ocular defects including microphthalmia, coloboma (a failure to properly close the optic fissure), corneal opacity, lens-cornea fusion, cataract, retinal folds, retinal detachment, glaucoma, and phthisis (a shrunken, atrophied eye)^75^. Regression of the hyaloid vasculature occurs in the first few weeks after birth in mouse and is a poorly understood process that involves macrophages secreting Wnt7b, a death signal for hyaloid endothelial cells^76,77^. Regression of the fetal vasculature also requires that retinal neurons upregulate VEGFR-2 to decrease levels of the endothelial survival factor VEGF^78^. Because *Myo10* is expressed in neurons, endothelial cells, macrophages, and melanocytes^26,29,79,80^, loss of *Myo10* could act in several ways, including stimulating an early hyperplasia of hyaloid cells, blocking recruitment of macrophages and apoptosis, and/or blocking filopodia-mediated signaling by Wnts or other factors.

Pigmented PFV, along with increased incidence of microphthalmia, have also been reported in a conditional KO of the Wnt receptor *Frizzled 5* ^81^. Pigmented PFV has been described in mice null for the *Arf* tumor suppressor gene^82^. The two obvious sources for pigmented cells in the eye would be the retinal pigmented epithelium, which is derived from the neuroectoderm and optic cup, and the melanocytes of the choroid, which are derived from the neural crest. KO of the guidance molecule *Ephrin-A5* also results in pigmented hyaloid vasculature and a high frequency of ocular defects, and in this case, the pigmented cells arrive shortly after birth and appear to be derived from choroidal melanocytes^83^. Recent work indicates that the initial cause of persistent hyaloid vasculature may be a defect or delay in the closure of the optic fissure^84^. Optic fissure closure involves both the fusion of epithelial sheets and apoptosis and shares many features with neural tube closure^85^. This observation, together with instances of failed eyelid closure, suggest that *Myo10* facilitates the fusion of epithelial sheets. Filopodia have been shown to help epithelial sheets zipper together during *Drosophila* dorsal closure^86^, so *Myo10*’s ability to induce filopodia may allow it to facilitate tissue fusion events in vertebrate development.

Although most cases of PFV in humans are unilateral and non-syndromic, approximately 10% are bilateral and associated with other abnormalities^87^. Several human syndromes with bilateral PFV have been identified, including Norrie disease, a disease due to defective Wnt signaling that is often associated with vascular defects, deafness, and developmental delay^88^. Because bilateral PFV is a prominent feature of the *Myo10* null phenotype, *MYO10* should be considered a candidate gene for syndromic bilateral PFV in humans.

### Other eye defects

In addition to 100% incidence of bilateral PFV, examples of several other eye defects were observed in *Myo10* null mice. These included examples of anophthalmia, microphthalmia, and defects in lens development. The MGI database indicates that in the two *Myo10* ^*m1J/m1J*^ mice that were examined, defects in optic fissure closure were present in two eyes, a thick and cloudy cornea was present in one, and a lens spot was present in one. Given the strong association between PFV and eye defects, some of the eye defects in *Myo10* null mice may be secondary to PFV. The 100% incidence of PFV in *Myo10* null mice makes them attractive models for investigating the mechanisms that underlie PFV and other eye defects.

### Webbed digits and kinked tails

All three *Myo10* null mutations characterized here exhibited ~50% penetrance of webbed digits, usually on the forelimbs. Vertebrate digits develop embryologically from fin-like structures, with the tissue between the digits removed by apoptosis. Apoptosis in the developing limb is induced in part by BMP signaling, and BMPs are also involved in skeletogenesis^89^. Although *Myo10* has not previously been implicated in apoptosis, *Myo10* is upregulated by BMP^26,27^ and binds to neogenin, a co-receptor for BMP^25^. Many *Myo10* ^*tm1d/tm1d*^ and *Myo10* ^*tm1a/tm1a*^ mice exhibited a small kink in the distal part of the tail. Although the basis for this defect is unknown, the mutation known as *kinked tail* is associated with a defect in notochord development^90^. Kinked tails along with craniorachischisis are also present in *looptail/Vangl2* mutants^91^ a component of the wnt-frizzled planar polarity pathway, and kinked tails are also present in deletions of *Sfrp2* (secreted frizzled-related protein 2)^92^.

### Hearing

The MyTH4-FERM myosins *Myo7a* and *Myo15a* localize to stereocilia and are required for hearing in human and mouse, raising the question of whether *Myo10* is required for hearing. *Myo10* mRNA is expressed in inner ear hair cells^93^, but unlike *Myo7a* and *Myo15a*, GFP-*Myo10* failed to localize to stereocilia^94^. The Jax database indicates that the two *Myo10* ^*m1J/m1J*^ homozygotes tested for auditory brainstem responses had elevated thresholds at all frequencies tested, although the size of this effect was not reported. In the young adult *Myo10* null mice examined here, hearing and balance appeared grossly intact based on the presence of the Preyer reflex and the absence of circling. It is possible that older *Myo10* mice exhibit a more severe hearing loss, but this is difficult to test in C57BL/6 mice since they already carry a *Cdh23* mutation that causes age-related hearing loss^95^.

### *Myo10* is not absolutely essential for mitosis or meiosis

An intriguing property of *Myo10* is that it can bind directly to microtubules via its MyTH4 domain^20–22^. Knockdown and dominant negative experiments in cell culture and *Xenopus* show that *Myo10* has several functions associated with microtubules, including spindle assembly and spindle orientation^20,41,96^. Because embryos can form in the complete absence of *Myo10* and some survive to adulthood and breed, our results demonstrate that *Myo10* is not absolutely essential for either mitosis or meiosis. This being said, loss of *Myo10* may have more subtle impacts on mitosis, meiosis, or spindle orientation, and these could potentially contribute to the phenotypes reported here. Other proteins may partially compensate for the loss of *Myo10*, especially given that *Myo10* and dynein have been shown to have partially overlapping functions in spindle orientation^41^. It is also possible that the requirements for *Myo10* in mitosis differ between cell culture, *Xenopus*, and mouse. Knockdown of *Myo10* in cell culture is also reported to have much more severe effects on mitosis in cancer cells than in normal cells^97^ due to *Myo10*’s role in clustering the excess centrosomes that are a hallmark of cancer cells.

### Conclusion and future directions

The analyses of the three *Myo10* null mouse strains reported here define the major phenotypes resulting from the complete loss of *Myo10*. Importantly, these analyses show that *Myo10* is required for production of normal numbers of filopodia *in vivo* and for normal vertebrate development. Future efforts should be directed at determining the molecular mechanisms by which *Myo10* functions in cellular processes such as filopodia formation and in organismal processes such neural development, pigmentation, and eye development. Our creation here of a conditional *Myo10* KO allele should greatly facilitate such investigations. Finally, it will also be important to investigate *Myo10’s* functions in signaling, especially given *Myo10*’s ability to bind receptors like DCC and neogenin, as well as growing evidence that filopodia help mediate signaling by BMPs, Wnts, and other morphogens^98^. The mice reported here provide key tools to investigate these and many other questions.

## Methods

### Mouse strain generation

All experiments and procedures involving mice at UNC, Stanford, and the NIH were approved and performed in accordance with the guidelines and regulations set forth by the UNC Institutional Animal Care and Use Committee (protocol 15–207), the Stanford Institutional Animal Care and Use Committee (protocol 23001), and the NIH Office of Animal Care and Use (protocol H-0269R1), respectively. The *Myo10* ^*tm1a*^ *, Myo10* ^*tm1c*^, and *Myo10* ^*tm1d*^ mouse strains used in this research project were generated from ES cell clone EPD0332_3_B02 obtained from the KOMP repository (www.komp.org) and generated by the Wellcome Trust Sanger Institute. These ES cells (*Myo10* ^*tm1a(KOMP)Wtsi*^, project CSD50217) were generated from parental JM8A1.N3 ES cells and are thus derived from a C57BL/6 N mouse. The *Myo10*-targeted ES cells use a promotor driven L1L2_Bact_P cassette that inserts an FRT site, a strong splice acceptor, an IRES, lacZ, a poly A site, a β-actin promotor, Neo resistance, and a FRT site in the intron immediately preceding exon 27, as well as loxP sites flanking exon 27. The *Myo10* ^*tm1a*^ ES cells were injected into blastocysts at the University of Michigan Transgenic Core Facility and chimeras were bred to C57BL/6 mice to get germline transmission. Chimeras were also bred to C57BL/6 mice expressing FLPo recombinase to delete the KO-first cassette and generate the *Myo10* ^*tm1c*^ conditional allele. Genotyping was used to confirm generation of the *Myo10* ^*tm1c*^ allele and to select breeders that did not carry FLPo. To generate a true genomic KO allele for *Myo10* (*Myo10* ^*tm1d*^) while maintaining the C57BL/6 background, mice carrying the *Myo10* ^*tm1c*^ conditional allele were crossed with C57BL/6 J β-actin global Cre deleter mice (strain #019099, Jax). Genotyping was used to confirm deletion of exon 27 and to select breeders that did not carry β-actin Cre. Sperm from C57BL/6 mice carrying the *Myo10* ^*tm1a*^ allele will be deposited in the Mutant Mouse Resource and Research Center following publication (www.mmrrc.org). *Myo10* ^*m1J*^ mice on a congenic C57BL/6 J background were obtained from Jax (strain #024583).

### Genotyping

The presence and zygosity of the *Myo10* ^*tm1a*^, *Myo10* ^*tm1c*^ and *Myo10* ^*tm1d*^ alleles were verified by PCR using genomic DNA purified from tissue collected at either the time of dissection or P10. Primers were designed for the locations denoted in Fig. 1B. PCR products were visualized by gel electrophoresis using 2% agarose containing 1X SYBR Safe DNA Gel Stain (Invitrogen, Catalog #S33102). Two primer sets were used for the *Myo10* ^*tm1a*^ allele: 30/31 identifies the lacZ cassette, while 24/29 determine zygosity – the *Myo10* ^*tm1a*^ allele produces a 567 bp band while the wild-type allele produces a 426 bp band. For the *Myo10* ^*tm1c*^ substrain, the 24/29 primer set will only produce a 265 bp band if the allele is present, whereas 24/25 will result in a 567 bp for *Myo10* ^tm1c^ and a 426 bp band for wild-type. Presence of the *Myo10* ^*tm1d*^ allele was verified using 24/27, which produces a 1400 bp band for wild-type and a 450 bp band for the *Myo10* ^*tm1d*^ allele. As a secondary measure of *Myo10* ^*tm1d*^ homozygosity, primer sets 26/27 were also used, which results in no product in mice homozygous for the *Myo10* ^*tm1d*^ allele. Genotyping primers used are as follows:

#24 (loxP2/3 Fwd): 5′-GTCCTCAGTCCCACATGACAA-3′;

#25 (loxP2/3 Rev): 5′-GAAGTCCCTTTAAGCAGCCC-3′;

#26 (Flank loxP3 Fwd): 5′-GGGAGCCGACATCAGTGTAG-3′;

#27 (Flank loxP3 Rev): 5′-ACCGCCCATATTACACCCCT-3′;

#28 (FRT1 Fwd): 5′-CTGGGTTTGGTCCTCAGTCC-3′;

#29 (FRT1 Rev): 5′-ATAGGAACTTCGGTTCCGGC-3′;

#30 (lacZ Fwd): 5′-GGTGAAGTGCCTCTGGATGT-3′;

#31 (lacZ Rev): 5′-CAAAAATCCATTTCGCTGGT-3′.

Two primer sets were designed for genotyping the *Myo10* ^*m1J*^ allele:

J-M10-E25d: 5′-CTTCGACGAGATCGACGAGTGCGTC-3′;

J-M10-E25-3-1: 5′-GTAAGGGTCCTCCTCGGATGAGTC-3′;

J-M10-E25-5: 5′-CAGGAGTTCTTGGAGTCGCTCAAC-3′;

J-M10-E25-3-2: 5′-CTCTTCGCCCGAAATCTCGCTC-3′.

To confirm homozygotes, a 5′-primer with an 8 bp deletion, J-M10-E25d, was used along with J-M10-E25-3-1; wild-type produces a 290 bp band while *m1J* produces nothing. The primers outside of 8 bp deletion, J-M10-E25-5 and J-M10-E25-3-2 are used to distinguish wild-type and heterozygotes. The difference between wild-type and *m1J* products (99 and 91 bp respectively) was detected using a 2% agarose gel.

### Immunoblotting

Because P5 mouse brain expresses both full-length and headless *Myo10*, it was used in immunoblotting to verify loss of *Myo10* in the *Myo10* ^*tm1a/tm1a*^ mice and their derivatives. Because *Myo10* protein undergoes rapid degradation, whole brain (including cerebellum) was weighed and immediately added to 1X Laemmli sample buffer (50 mM Tris, pH 7.4, 2% SDS, 6% glycerol, 0.1 M DTT and 0.25% bromophenol blue) at 500 μL/10 mg wet brain weight and boiled for 15 min. Samples were thoroughly aspirated to dissociate the tissue and the lysates were centrifuged at 20,000 g for 30 min at RT. Serial dilutions of each lysate was subject to SDS-PAGE using a Bolt 4–12% Bis-Tris Plus gel (ThermoFisher). Final loading volumes were adjusted for immunoblot based on quantification of the actin band from Coomassie blue stained gels. After SDS-PAGE, lysates were transferred to nitrocellulose at room temperature using a Hoefer TE22 transfer tank containing Transfer Buffer (20 mM Tris, 200 mM Glycine, 20% Methanol, and 0.1% SDS) set to 100 V for 1.5 hrs, followed by blocking for 30 min in PBS containing 10% dried milk powder. Membranes were then incubated for 30 min in PBS containing 5% dried milk and 0.25% Tween 20 plus 1 μg/mL rabbit anti-*Myo10* (Sigma #HPA024223; Lot# A60694) and 1 μg/mL mouse anti-actin (Abcam #ab3280), followed by three 5 min washes in PBS with 0.5% Tween 20. Membranes were then incubated for 30 min in PBS containing 5% dried milk, 0.25% Tween 20, 0.05% SDS with IRDye 680LT goat anti-rabbit IgG and IRDye 800CW Donkey anti-mouse IgG (LI-COR #926–68021 and #926-32212), both at 0.1 μg/mL. Following three 5 min washes, blots were imaged on a LI-COR Odyssey CLx infrared imaging system. Brightness/Contrast for the *Myo10* (700 nm) and Actin (800 nm) channels was uniformly adjusted across the full blot using LI-COR ImageStudio software, then exported for final processing in ImageJ. The actin channel was displayed in red and overlaid onto the *Myo10* channel, which was converted to inverted gray scale.

### Embryo dissections

Timed pregnancies were monitored by plug check, with noon on the morning a plug was present defined as E 0.5. Pregnant females were euthanized by CO_2_ asphyxiation. Intact uterine horns were removed from the abdominal cavity and placed in a 10-cm petri dish containing PBS. After separating embryos from each other, the uterine wall, Reichert’s membrane, visceral yolk sac and placenta were trimmed away. Tail tissue was taken to verify genotype. Whole embryos were then placed in 4% paraformaldehyde overnight at 4 °C prior to imaging.

### Preparation of eye cups and retinal flat mounts

Due to the high level of exencephaly, it was difficult to reliably obtain *Myo10* null pups with littermate controls from het-het matings. We therefore bred *Myo10* ^*tm1d/tm1d*^ males with *Myo10* ^+/tm1d^ females to obtain greater numbers of *Myo10* ^*tm1d/tm1d*^ pups and compared these to *Myo10* ^+/+^ controls from separate matings. To obtain eyes, P5 pups were euthanized by decapitation. The eyes were immediately enucleated and incubated in 4% paraformaldehyde in PBS for 1 hr at RT. After trimming excess scleral connective tissue, the cornea and iris was removed using spring scissors. To remove the lens, four small incisions were made across the corneoscleral divide, which prevents retinal detachment when removing the lens from the eyecup. Once the lens and elements of the vitreous were removed, the four incisions (90° to each other about the eyecup) were extended through the sclera to the optic nerve, resulting in a retina with a 4-petal shape that can be flat-mounted. A round-blade scalpel was used to sever the retina from its junction point with the cornea. The sclera was then removed by gently peeling the retina off and severing the optic nerve.

Retinas were then permeabilized in 0.5% Triton X-100 in PBS for 20 min at RT, followed by 3 washes with PBS. Retinas were blocked in 5% bovine serum albumin (Sigma #A4503) in PBS for 4 hrs at RT, followed by overnight incubation at 4 °C with 0.3 μg/mL of rat anti-PECAM-1 (BD Biosciences #550274) in 5% BSA/PBS. After 3 washes with 0.1% Triton X-100 in PBS and 2 washes with PBS, retinas were incubated with 2 μg/mL of Alexa Fluor 488-conjugated donkey anti-rat IgG secondary antibody (Invitrogen #A21208) in 5% BSA/PBS and counterstained with 13.2 nM Alexa Fluor 568-labeled phalloidin and 22 μM DAPI (Molecular Probes #A12380 and #D3571). Retinas were then flat-mounted with “Gelvatol,” a semi-hardening mounting media: 10% polyvinyl alcohol (Sigma #P8136), 20% glycerol (Sigma #P5516) and 0.2% N-propyl gallate (Sigma #02370) in 0.1 M Tris, pH 8.5.

### Hematoxylin and eosin staining of embryo sections

Mouse embryos at E16.5 were fixed in 4% PFA overnight, embedded in paraffin, and 5 μm sections were cut and mounted on slides. The sections were deparaffinized by xylene and stained by H&E solution. Nanozoomer 2.0 RS scanner (Hamamatsu) was used to image the slides.

### Image Acquisition and Image Analysis

An Olympus FluoView FV1200 confocal system was used for fluorescent images of retinal flat mounts. Tiled Z-stack images of entire retinas were captured with a 20X (0.75 NA) lens. To visualize filopodia, Z-stacks were collected with a 60X (1.35 NA) oil immersion lens and a Z step of 0.2 μm, then tiled to assemble an image of the entire vascular front along each petal of a flat-mounted retina. Maximum intensity projections for both 20X and 60X tiled images were then stitched using the Paired Stitching plug-in for FIJI/ImageJ^99^. Images of embryos, tails, and eyes were acquired using a Leica M205 FA equipped with a MicroPublisher CCD camera (Q Imaging).

### Statistics

Error bars in figures and ± symbols indicate standard deviations unless indicated otherwise. P-values were calculated using two-tailed Student’s t-tests.

### Data Availability Statement

The datasets generated during and/or analyzed during the current study are available from the corresponding author upon request.

## Electronic supplementary material

Supplementary Figure 1
