# Supplementary material for: Myosin-X knockout is semi-lethal and demonstrates that myosin-X functions in neural tube closure, pigmentation, hyaloid vasculature regression, and filopodia formation
Source: Sci Rep. 2017 Dec 11;7:17354. doi: 10.1038/s41598-017-17638-x (PMC5725431; doi:10.1038/s41598-017-17638-x)
Supplement: Supplementary file 1 — Supplementary Figure 1 [file 41598_2017_17638_MOESM1_ESM.pdf]

Supplementary Information for:

Myosin-X knockout is semi-lethal and demonstrates that myosin-X functions in neural tube closure, pigmentation, hyaloid vasculature regression, and filopodia formation

Ernest G. Heimsath, Jr., Yang-In Yim, Mirna Mustapha, John A. Hammer, and Richard E. Cheney

A

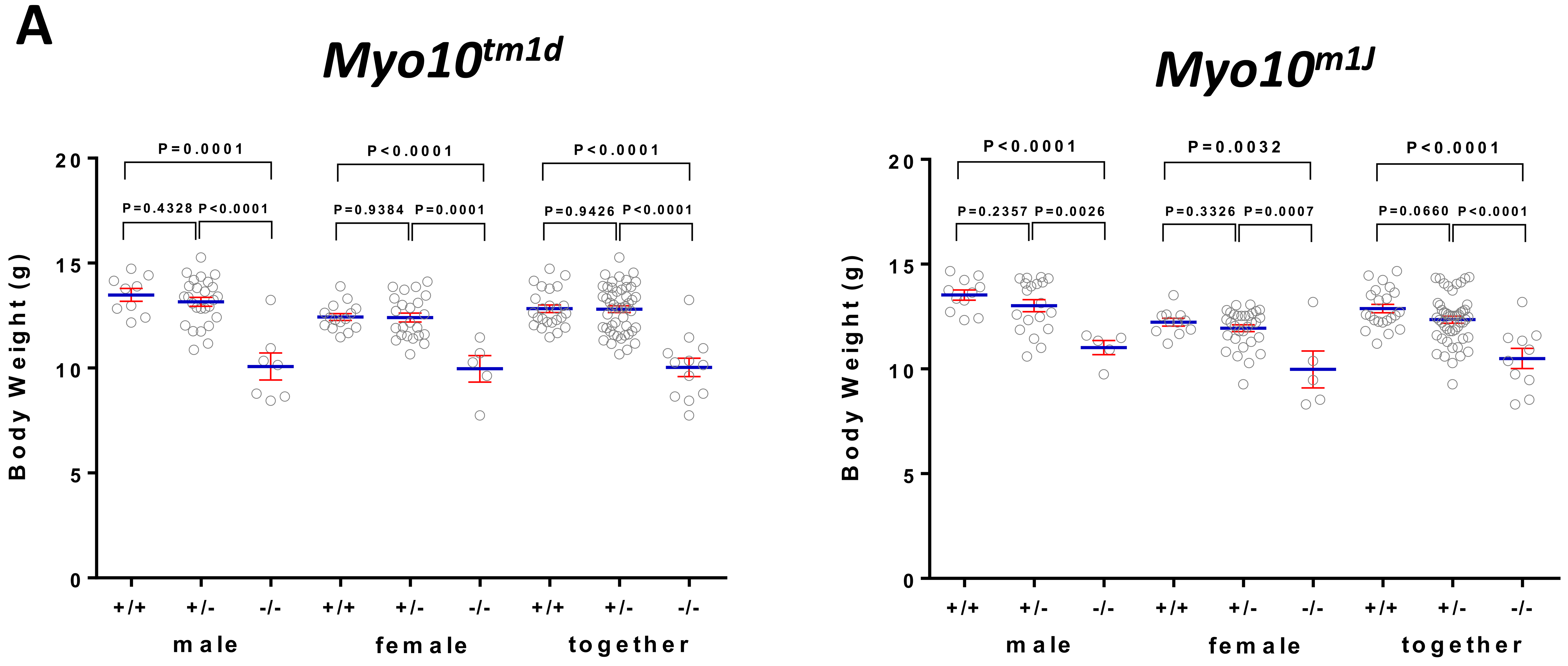

B

	Genotype	Mean weights (g +/- SEM (n))		
		male	female	together
Myo10^{tm1d}	+/+	13.48 +/- 0.31 (9)	12.43 +/- 0.16 (15)	12.83 +/- 0.18 (22)
	+/-	13.16 +/- 0.22 (26)	12.41 +/- 0.21 (23)	12.81 +/- 0.16 (49)
	-/-	10.07 +/- 0.64 (7)	9.96 +/- 0.63 (5)	10.03 +/- 0.44 (12)
Myo10^{m1J}	+/+	13.53 +/- 0.24 (11)	12.23 +/- 0.18 (11)	12.88 +/- 0.20 (22)
	+/-	13.02 +/- 0.29 (19)	11.94 +/- 0.16 (31)	12.35 +/- 0.16 (50)
	-/-	11.02 +/- 0.34 (5)	9.98 +/- 0.89 (5)	10.50 +/- 0.48 (10)

Supplementary Figure 1. The mean weights of *Myo10$^{tm1d/tm1d}$* and *Myo10$^{m1J/m1J}$* null mice are slightly smaller than *Myo10$^{+/+}$*. (A) The weights of the wild type, heterozygous, and null mice produced in colonies of *Myo10^{tm1d}* and *Myo10^{m1J}* mice were determined at P28. This data is from heterozygous matings of colonies maintained at the NIH. Data was plotted for males, females, and males and females together. P-values are from unpaired t-tests and P <0.05 is defined as significant. **(B)** Table of mean weight, SEM and n values for *Myo10$^{tm1d/tm1d}$* and *Myo10$^{m1J/m1J}$* mice represented in (A).
